# Editorial: Behavior and heat stress

**DOI:** 10.3389/fvets.2023.1219955

**Published:** 2023-06-14

**Authors:** Cristiane Gonçalves Titto, Fabio Luis Henrique, Messy Hannear de Andrade Pantoja, Cihan Çakmakçı, Priscila dos Santos Silva

**Affiliations:** ^1^Faculty of Animal Science and Food Engineering, University of São Paulo, Pirassununga, Brazil; ^2^Faculty of Veterinary Medicine, University of the Republic, Montevideo, Uruguay; ^3^Department of Agricultural Biotechnology-Animal Biotechnology, Faculty of Agricuture, Van Yüzüncü Yil University, Van, Türkiye; ^4^Anisio Teixeira College, Feira de Santana, Brazil

**Keywords:** acclimation, adaptation, behavioral response, climate change, temperature, thermoregulation

Relating the environment to the quality of life of animals becomes essential when the goal is to achieve welfare. Climatic variations, highlighted mainly by the increase in temperature in recent years, are directly associated with the search for thermal balance for several species to avoid situations of heat stress, which can result in cellular and molecular dysfunctions, thermoregulatory and behavioral disorders, and consequently reduce the health of companion, sports, working, and production animals.

This Research Topic is composed of eight original articles and three reviews, which present arguments through the advancement of tools and technologies for measuring animal temperature; physiological, biochemical, and behavioral changes; handling practices and facilities; and economic advantages, which aim to validate the environmental impact on the thermal comfort zone of these individuals.

These changes can be observed in companion animals, such as dogs and cats, that share the same environment as humans. Palestrini et al. demonstrate through their data that the behavior of these animals is altered with the variation of the ambient temperature. Even without showing an increase in aggressive behavior, both species become more active with milder temperatures and sleep more when there is a greater variation, both upward and downward.

Production animals are constantly studied in this line of research because of their dependence on the control of meteorological variables to achieve high performance. As described by Ragab et al. in their study of rabbits, genetic factors can affect an individual's susceptibility to environmental conditions.

To demonstrate this interaction with the environment, Govindasamy et al. working with different genotypes of pigs, found alterations in physiological, behavioral, and biochemical parameters through measurements of temperature (rectal and skin surface), respiration rate, heart rate, behavior, and hormones measured by blood in a specific environment, and different pig breeds may respond differently to seasonal changes. Gómez-Prado et al. in turn, pointed out the main thermoregulation mechanisms used for this heat exchange between pigs and the environment, addressing concepts for situations not only of hyperthermia but also of hypothermia and highlighting the measurement of temperature using infrared thermography.

Non-invasive technologies are increasingly being used as alternatives for monitoring the body temperature of animals, given the need to validate their use between species, and in comparison with internal body temperatures. For example, Verdegaal et al. studied the applicability of monitoring skin surface temperature as a thermoregulatory response metric in endurance horses during field exercises, as well as the relationship between skin surface temperature and core temperature as measured by gastrointestinal temperature. They found that skin surface temperature monitoring does not provide a reliable proxy for the thermoregulatory response in horses, due to many factors that can modulate skin surface temperature without directly affecting core temperature, and revealed important inter-individual differences in skin surface and core temperature. Mota-Rojas et al. who also discuss the use of infrared thermography, highlight the great usefulness of the tool in measuring the body temperature of newborn ruminants, relating moments of hyperthermia with the development of muscle and adipose cells in these animals and the action of metabolic routes for the utilization of these energy reserves for the activation of thermoregulation pathways. Similarly, Napolitano et al. showed that birth weight is a factor that alters the thermoregulation of water buffalo calves during the first days of life, but that colostrum ingestion can be a compensatory action for better development in such situations, making the thermoregulatory mechanism more efficient.

By studying dairy cows, Shu et al. sought to improve the technique for measuring ocular temperature under heat stress, which is often found in high-performance herds in dairy production. Their study results suggested that infrared thermography can provide a non-invasive and practical approach for assessing heat stress in dairy cows. In addition, the results provided useful recommendations regarding the optimal direction of image acquisition, highlighting the regions of the eye that most effectively reflect thermoregulatory responses in these instances.

In relation to thermoregulation and behavior, the facilities, and methods of handling livestock in production systems are crucial points to discuss. Studying dairy cows, Santos et al. traced the thermoregulatory profile of these animals in tropical environments, mainly discussing environmental aspects and installations that can alter the thermal equilibrium point in pasture production systems. Rodríguez-González et al. in their study, addressed transport management as a possible stress trigger for water buffalos and sought to relate different temperature measurements to represent these values for meat-producing animals. Finally, Maia et al. showed economic advantages when using a shade design to offer confined beef cattle a better thermoregulatory condition, relating their thermal comfort zone within the production system and resulting in increased production rates.

## Author contributions

The editorial board of this Research Topic was composed by the CT as main editor. MP, CÇ, FH, and PS as guest editors working in the organization and review of articles. The editorial article was written by the FH and revised by CT, MP, CÇ, and PS. All authors contributed to the article and approved the submitted version.

